# Impact of NT-proBNP reduction on recurrence after cryoballoon pulmonary vein isolation and left atrial roof ablation in persistent atrial fibrillation

**DOI:** 10.1007/s00380-025-02559-x

**Published:** 2025-06-06

**Authors:** Ryohei Nomura, Kanae Hasegawa, Toshihiko Tsuji, Moe Mukai, Machiko Miyoshi, Naoto Tama, Hiroyuki Ikeda, Kentaro Ishida, Hiroyasu Uzui, Hiroshi Tada

**Affiliations:** Department of Cardiovascular Medicine, Faculty of Medical Sciences, University of Fukui, 23-3, Matsuokashimoaizuki, Eiheiji-cho, Yoshida-gun, Fukui, 910-1193 Japan

**Keywords:** Cryoballoon ablation, LA roof ablation, NT-proBNP, Persistent atrial fibrillation, Recurrence

## Abstract

Pulmonary vein (PV) isolation by catheter ablation is a widely used curative therapy for atrial fibrillation (AF). However, in patients with persistent AF (PeAF), long-term outcomes are poor when PV isolation is performed alone. Although left atrial (LA) roof ablation is sometimes added to PV isolation, its effectiveness and predictors of success remain unclear. To identify predictors of arrhythmia recurrence in patients with PeAF undergoing LA roof ablation and PV isolation using a cryoballoon catheter. A retrospective assessment of LA roof ablation and PV isolation using a cryoballoon was performed in 65 consecutive patients with PeAF. The median age of the patients was 69 years [Q1:61, Q3:75]; 55% were female, and 49% had longstanding PeAF. The complete LA roof block success rate using a cryoballoon was 92.3%, with no esophagus-related complications. The 1-year post-ablation arrhythmia-free rate was 83.3%. Atrial arrhythmia recurrence was more common within the 3-month blanking period. The N-terminal prohormone of brain natriuretic peptide (NT-proBNP) reduction rate ≥60.7% and sinus rhythm at 1-month post-ablation, and no arrhythmia during the 3-month blanking period strongly predicted arrhythmia-free status at 1 year post-ablation. In patients with PeAF who underwent LA roof ablation and PV isolation using a cryoballoon, the arrhythmia-free rate was high. The NT-proBNP reduction rate at 1-month post-ablation may serve as a simple and potentially useful predictor of procedural success.

**Supplementary Information:**

The online version contains supplementary material available at 10.1007/s00380-025-02559-x.

## Introduction

Pulmonary vein (PV) isolation by catheter ablation is a widely performed curative therapy for atrial fibrillation (AF), with cryoballoon-based PV isolation now the first-choice treatment for paroxysmal AF [1–4]. However, in patients with persistent AF (PeAF), the long-term AF recurrence rate remains relatively high and unsatisfactory [5–7]. The efficacy of adding left atrial (LA) roof ablation to PV isolation in patients with AF has recently been investigated, but the use of radiofrequency energy or cryoballoons for this purpose remains controversial [5, 8, 9]. Consequently, the benefit of adding LA roof ablation in patients with PeAF has yet to be fully clarified. Factors such as AF duration and plasma brain natriuretic peptide (BNP) levels have been identified as predictors of long-term arrhythmia recurrence [10, 11]. However, no study has specifically evaluated predictors of arrhythmic recurrence after LA roof ablation and PV isolation in patients with PeAF. This study aimed to address this gap by identifying predictors in patients with PeAF undergoing these procedures with a cryoballoon.

## Materials and methods

### Study population

This retrospective cohort study included 65 consecutive patients with PeAF who underwent LA roof ablation and PV isolation using a single 28-mm cryoballoon (Arctic Front Advance or Artic Front Advance PRO, Medtronic Inc., Minneapolis, MN, USA) from October 2021 to May 2024 at the University of Fukui. AF was classified according to the latest guidelines [1]. All patients provided written informed consent for the procedure, and the study protocol was approved by the Research Ethics Committee of the University of Fukui (20160040). This study complied with the principles of the Declaration of Helsinki. Data supporting the findings of this study are available from the corresponding author upon reasonable request.

### PV isolation and LA roof ablation using cryoballoon and mapping

Surface electrocardiogram (ECG) and bipolar intracardiac electrograms were continuously monitored and stored on a computer-based digital recording system (EP-WorkMate, Abbott, IL, USA). A 7-Fr 20-pole three-site mapping catheter (BeeAT, Japan-Lifeline Co., Ltd., Tokyo, Japan) was inserted through the right jugular vein for pacing, recording, and internal cardioversion. The procedure was performed under moderate sedation with dexmedetomidine and thiopental and under uninterrupted anticoagulation therapy with dabigatran. Heparin (100 IU/kg body weight) was administered immediately after venous access, and heparinized saline was additionally infused to maintain an activated clotting time of 300–350 s.

A single transseptal puncture was performed using a radiofrequency needle (Baylis Medical, Inc., Montreal, QC, Canada) and an 8-Fr long sheath (SL0, AF Division, Abbott, IL, USA), guided by fluoroscopy and intracardiac ultrasound (Acunav, Ultrasound Catheter, Biosense Webster, CA, USA). The transseptal sheath was exchanged over a guidewire for a 15-Fr steerable sheath (FlexCath Advance, Medtronic, MN, USA). A spiral mapping catheter (Achieve, Medtronic, MN, USA) was used to advance the cryoballoon into the PV for support and map the PV potentials. After the confirmation of complete sealing of the PV with a 28-mm cryoballoon using contrast medium injection or changing the pressure waveform [12], a freeze cycle of maximal 180 s was applied, and the dose was adjusted. No additional applications were performed after isolation. To avoid right phrenic nerve injury, diaphragmatic electromyography was monitored during cryoballoon application to the right PVs. If the balloon temperature reached −55 degrees or the amplitude of electromyography significantly decreased, or the motion of the diaphragm during upper abdominal breathing decreased, the freezing was terminated with passive deflation. If PV isolation was not achieved with the cryoballoon, additional touch-up ablation was performed using an irrigated-tip catheter (FlexAbility, Abbott, IL, USA). The endpoint was defined as electrical PV isolation, which was verified using a 20-mm Achieve catheter.

Additional LA roof ablation was performed. The Achieve catheter was placed deep in the right superior PV (RSPV) to stabilize the cryoballoon, with the distal balloon freezing surface oriented towards the LA roof. The first application was performed in close proximity to the balloon during the RSPV isolation by slight counter-clockwise rotation of the steerable sheath in combination with a slight retraction of the sheath and incremental advancement of the cryoballoon. Sequential freezing was performed along the LA roof in an overlapping manner (Online Resource 1). If the application from the RSPV side was insufficient, additional cooling was performed by inserting a 20-mm Achieve catheter into the left superior PV (LSPV). The freezing dose was 2–4 min.

If AF persisted after cryoballoon ablation and additional LA roof ablation, internal electrical cardioversion was performed to restore sinus rhythm. During atrial pacing at the distal coronary sinus, a detailed voltage map of the LA was created using a high-density mapping system (Rhythmia, Boston Scientific, Natick, MM, USA) and a 64-minielectrode basket mapping catheter (Orion, Boston Scientific). Bipolar electrograms were obtained. Low-voltage and scar areas were defined as areas with <0.5 and <0.1 mV, respectively. The PV isolation and roof line blocks were defined as the scar areas in the area of interest. The conduction block in the LA roof was also examined via activation mapping using a high-density mapping system during atrial pacing at the distal coronary sinus.

Subsequently, if atrial tachycardia (AT) and atrial flutter were induced by atrial pacing or if these atrial arrhythmias were documented clinically, additional radiofrequency (RF) ablation was performed.

### Roof line measurements

**Fig. 1:**
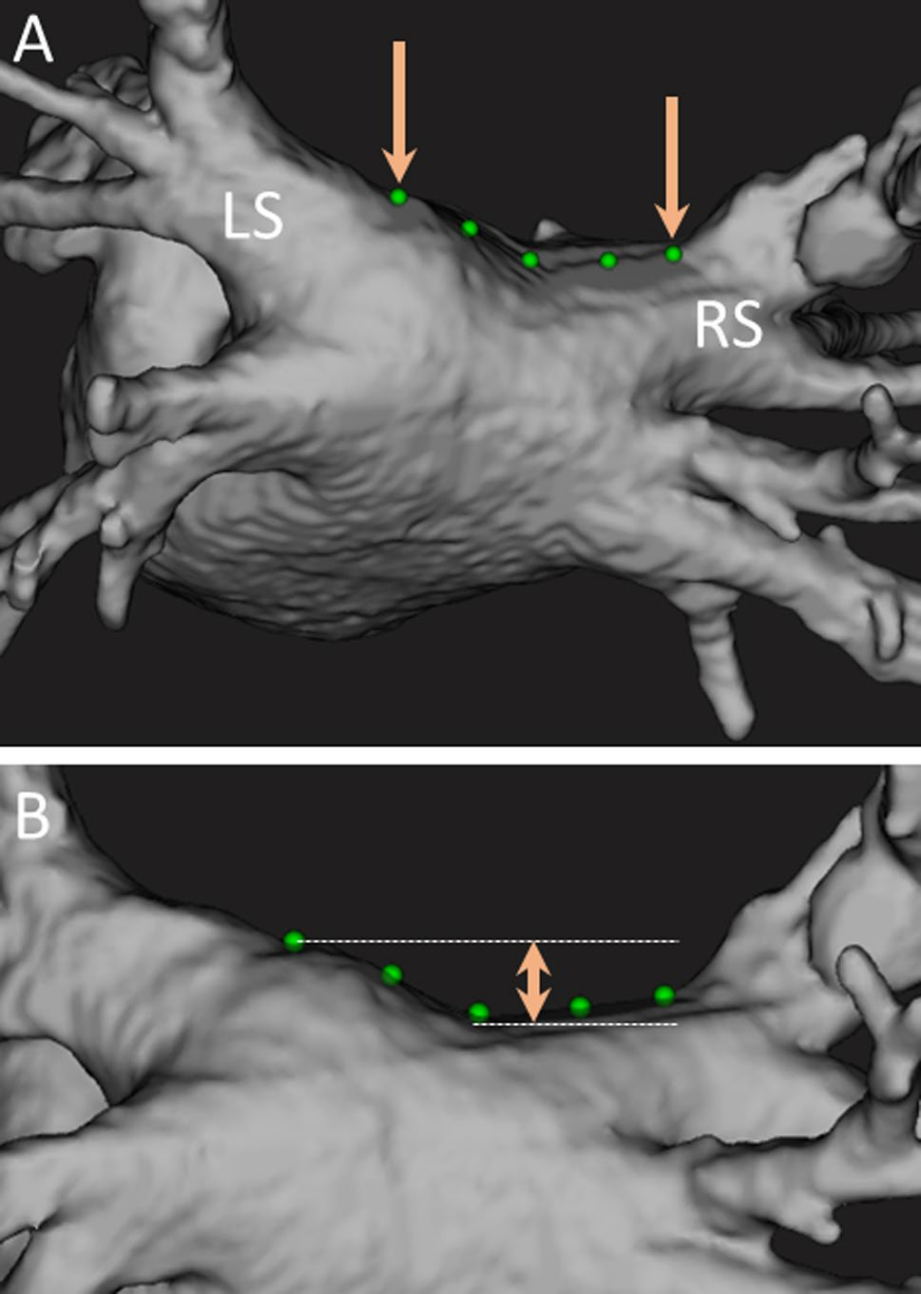
Measurement for roof line length (**A**) and height (**B**).** A** The inflection points of the left and right superior pulmonary veins and left atrium are first identified as indicated by the arrows. Three points on the LA roof are chosen between these inflection points, and the distance drawn between them is measured as the roof line length. **B** The height of the roof line is determined by measuring the distance between its highest and lowest points on the posterior anterior view CT imaging. LS, left superior pulmonary vein; RS, right superior pulmonary vein; LA, left atrial; CT, computed tomography

All patients underwent preprocedural cardiac computed tomography (CT) (SOMATOM Force, SIEMENS, Erlangen, Germany), and the CT imaging was analyzed using a workstation. The inflection points of the LSPVs, RSPVs, and LA were first identified (arrows in Fig. 1A). Three points on the LA roof were selected between these inflection points, and the distance between them was measured as the roof line length (Fig. 1A). The height of the roof line was determined by measuring the distance between its highest and lowest points on posterior anterior view CT (Fig. 1B).

### Follow-up

Patients underwent continuous in-hospital ECG monitoring for 3–5 days after the procedure. The first outpatient clinic visit was performed 1 month after the procedure. The subsequent follow-up visits consisted of clinical interviews, 3-min ECG, and/or 24-h Holter monitoring every 2–3 months at our cardiology clinic. Blood samples for plasma N-terminal Prohormone of BNP (NT-proBNP) levels were obtained at the time of hospital admission (specifically, one day prior to the ablation procedure) and again at the 1-month follow-up visit. Successful ablation was defined as the absence of AF or AT lasting >30 s for 1 year, excluding a blanking period of 3-month post-ablation.

### Statistical analysis

Continuous variables are presented as medians and interquartile ranges, and categorical data are presented as numbers and percentages. Univariate analyses of categorical variables were performed using the Fisher’s exact test. To avoid the assumption of normality, univariate analyses of the continuous variables were performed using the Wilcoxon rank-sum test. Multivariable logistic regression models were constructed, including the following factors: AF duration, the reduction rate of NT-proBNP level at 1-month post-ablation (defined as the NT-proBNP level at the preprocedure minus the NT-proBNP level at 1-month post-ablation, divided by the preprocedure NT-proBNP level; ΔNT-proBNP_1 month_), sinus rhythm at 1-month post-ablation (Analysis model 1), and no recurrence (AF/AT) within the 3-month blanking period (Analysis model 2). Statistical analyses were performed using the SPSS software version 26 (IBM Corp., Armonk, NY, USA). A receiver-operating characteristic (ROC) curve was created using EZR (Saitama Medical Center, Jichi Medical University, Saitama, Japan), which is a graphical user interface for R (The R Foundation for Statistical Computing, Vienna, Austria) [13].

## Results

### Patient characteristics

The patient characteristics of the 65 patients were as follows: median age of patients was 69 [Q1: 61, Q3: 75] years, 55% were female, and 49% was longstanding PeAF, defined as continuous AF > 12 months. Median LA diameter was 44 [40, 46] (Table 1).

**Table 1:** Baseline characteristics of the study cohort

Age, years	69 [61, 75]
Female gender, *n* (%)	36 (55)
Body mass index, kg/m^2^	24 [21, 27]
AF duration, months	11 [6, 30]
Longstanding PeAF, *n* (%)	32 (49)
CHADS_2_ score	1 [1, 2]
CHA_2_DS_2_-VASc score	2 [2, 4]
Congestive heart failure, *n* (%)	20 (31)
Hypertension, *n* (%)	45 (69)
Diabetes mellitus, *n* (%)	16 (25)
Prior stroke or transient ischemic attack, *n* (%)	4 (6)
NT-proBNP, pg/mL	833 [499, 1273]
Antiarrhythmic drugs	
None, *n* (%)	51 (78)
Class I, *n* (%)	2 (3)
Amiodarone, *n* (%)	10 (15)
Bepridil, *n* (%)	0 (0)
Verapamil, *n* (%)	2 (3)
Concomitant medications	
Loop diuretics, *n* (%)	10 (15)
Thiazide diuretics, *n* (%)	2 (3)
Vasopressin V2 receptor antagonists, *n* (%)	1 (2)
Mineralocorticoid receptor antagonist, *n* (%)	8 (12)
SGLT2 inhibitors, *n* (%)	14 (22)
Echocardiographic parameters	
Left ventricular ejection fraction, %	60 [50, 64]
Left atrial diameter, mm	44 [40, 46]
Left atrial volume, mL	84.6 [73.6, 92.4]
Left atrial volume index, mL/m^2^	47.4 [41.3, 56.0]

Values are expressed as *n* (%) or median [first and third quartiles]

*AF* atrial fibrillation, *PeAF* persistent atrial fibrillation, *SGLT2* sodium-glucose cotransporter 2, *NT-proBNP* N-terminal prohormone of brain natriuretic peptide

Among the 65 patients, 14 (22%) were receiving sodium–glucose cotransporter 2 (SGLT2) inhibitors at baseline. One additional patient initiated SGLT2 inhibitor therapy after ablation, resulting in 15 patients (23%) at the 1-month follow-up. The number of patients receiving loop diuretics decreased from 10 (15%) at baseline to 9 (14%) at the 1-month follow-up, while the use of other diuretics remained unchanged during that period.

### PV isolation and LA roof ablation using cryoballoon

**Fig. 2:**
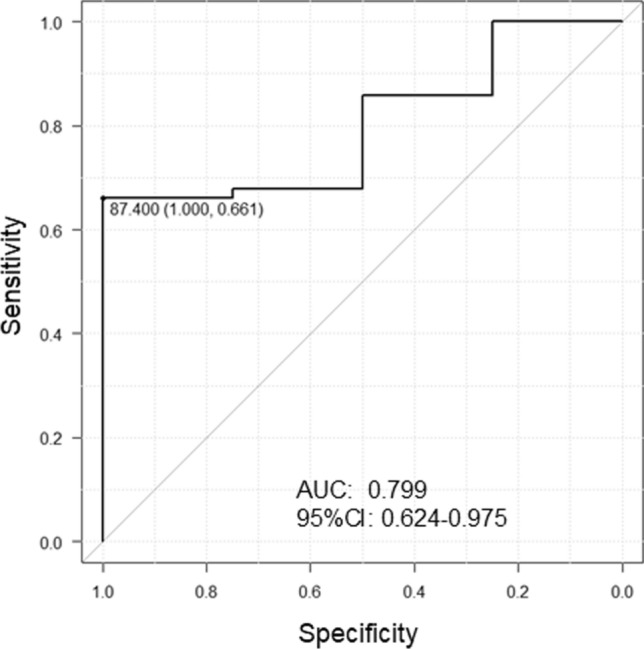
Receiver operating characteristic (ROC) curve for complete roof line block and the left atrial volume. The optimal cutoff value of the LA volume is 87.4 mL with a sensitivity of 66.1% and specificity of 100%. AUC, area under the curve; CI, confidence interval

PV isolation was successfully achieved in 58 (89.2%) using cryoballoon only. Among the remaining 7 patients, 8 PVs (RSPV in 1, right inferior PV in 6, and left inferior PV in 1) required touch-up ablation. After PV isolation, LA roof ablation with complete block was successfully achieved in 60 patients (92.3%). Cryoballoon ablation data and LA roof anatomical measurements were presented in Table 2. The total freezing duration and number of cryoballoon application for LA roof were 960 [955, 1200] s and 5 [4, 6] points. The median nadir balloon temperature −44 [−47, −41] °C. In patients who achieved complete block of the LA roof (complete roof block), the number of cryoballoon applications was lower and the LA volume was smaller than in those who did not achieve complete roof block (both *P* < 0.05). In the ROC curve, the LA volume < 87.4 mL predicted the complete roof block with an area under the curve (AUC) of 0.799 (*P* = 0.018; Fig. 2). Patients with complete roof block tended to have a shorter AF duration, shorter roof line length and height, and smaller LA diameter and LA volume index than those without block, although these differences were not statistically significant. In addition to PV isolation and LA roof ablation, a cavotricuspid isthmus block line was created in 15 patients (23.1%), a mitral isthmus block line in 2 (3.1%), and isolation of the superior vena cava in 1 (1.5%).

**Table 2:** Roof line ablation with or without complete block

	All	Complete block	Incomplete block	*P* value
	(*n* = 65)	(*n* = 60)	(*n* = 5)	
AF duration, months	11 [6, 30]	11 [5, 28]	26 [10, 42]	0.194
Longstanding PeAF, *n* (%)	32 (49)	29 (48)	3 (60)	0.485
Total freezing time, s	960 [955, 1200]	960 [940, 1200]	960 [960, 960]	0.241
Target freezing time per application, s	240 [240, 240]	240 [240, 240]	240 [120, 240]	0.710
Median freezing temperature, °C	−44 [−47, −41]	−44 [−47, −41]	−42 [−49, −40]	0.711
Number of applications, points	5 [4, 6]	5 [4, 6]	8 [6, 10]	0.012
Roof line length, mm	40.0 [33.9, 45.8]	39.9 [33.9, 45.3]	46.0 [33.9, 50.7]	0.393
Height of roof line, mm	10.3 [8.1, 12.8]	10.3 [8.0, 12.7]	13.9 [6.45, 18.3]	0.098
Left atrial diameter, mm	44 [40, 46]	43 [40, 46]	47 [43, 51]	0.092
Left atrial volume, mL	84.6 [73.6, 92.4]	84.1 [73.3, 92.0]	92.4 [88.0, 133.9]	0.046
Left atrial volume index, mL/m^2^	47.4 [41.3, 56.0]	46.7 [41.3, 55.9]	57.7 [43.8, 84.7]	0.175

Values are expressed as *n* (%) or median [first and third quartiles]

*AF* atrial fibrillation, *PeAF* persistent atrial fibrillation

### Clinical outcomes

Single-procedure freedom from atrial arrhythmia at 12 months (median follow-up period, 174 [109, 284] days) was 83.3% (49.2% on antiarrhythmic drug therapy). During follow-up, 10 patients had a recurrence of atrial arrhythmias, including AF in 7 patients and AT in 3. A detailed comparison between patients with and without arrhythmia recurrence is presented in Table 3. The patients with arrhythmia recurrence had longer AF duration (*P* = 0.003) and more frequent long-standing PeAF (*P* = 0.006) than those without recurrence. LA size and baseline NT-proBNP levels were comparable between the patients with and without arrhythmia recurrence. However, the NT-proBNP level at 1-month post-ablation (*P* = 0.006) was higher, and ΔNT-proBNP_1 month_ (*P* < 0.001) was lower in the arrhythmia recurrence group than in the no recurrence group. Among patients with arrhythmia recurrence, 60% had recurrence on follow-up ECG at 1-month post-ablation (*P* = 0.001), and 80% had arrhythmia recurrence within the 3-month blanking period (*P* < 0.001), both significantly more frequently than in those with no arrhythmia recurrence.

**Table 3:** Univariable analysis for risk of recurrence of atrial arrhythmia

	Recurrence (−)	Recurrence (+)	*P* value
	(*n* = 55)	(*n* = 10)	
Age, years	70 [61, 75]	69 [59, 77]	0.906
Female gender, *n* (%)	12(22)	2(20)	0.633
Body mass index, kg/m^2^	24 [21, 27]	26 [22, 29]	0.317
AF duration, months	10 [5, 21]	33 [22, 57]	0.003
Longstanding persistent AF, *n* (%)	23 (42)	9 (90)	0.006
CHADS_2_ score	1 [1, 2]	2 [1, 3]	0.669
CHA_2_DS_2_-VASc score	2 [2, 4]	3 [1, 5]	0.838
Echocardiographic parameters			
Left ventricular ejection fraction, %	60 [49, 63]	65 [61, 67]	0.086
Left atrial diameter, mm	44 [40, 46]	45 [41, 46]	0.561
Left atrial volume, mL	84.1 [72.9, 92.1]	88.1 [78.9, 102.5]	0.341
Left atrial volume index, mL/m^2^	46.7 [40.8, 56.2]	51.5 [43.0, 58.5]	0.428
Catheter ablation			
PV isolation with touch-up ablation, *n* (%)	5 (9)	2 (20)	0.292
Roof line with complete block, *n* (%)	52 (95)	8 (80)	0.166
Additional ablation to PV isolation and roof line, *n* (%)	16 (29)	2 (20)	0.713
Antiarrhythmic drugs at follow-up period			
None, *n* (%)	30 (55)	3 (30)	0.139
Class I, *n* (%)	2 (4)	1 (10)	0.399
Amiodarone, *n* (%)	11 (20)	5 (50)	0.057
Bepridil, *n* (%)	12 (22)	1 (10)	0.356
NT-proBNP			
Baseline, pg/mL	862 [536, 1,525]	633 [400, 956]	0.164
1-month post-ablation, pg/mL	174 [110, 371]	482 [332, 864]	0.006
Reduction rate of NT-proBNP at 1-month post-ablation, %	79.4 [63.7, 87.2]	30.6 [−14.5, 51.6]	<0.001
Patients with NT-proBNP levels lower than baseline values at 1-month post-ablation, *n* (%)	53 (96)	6 (60)	0.004
Sinus rhythm at 1-month post-ablation, *n* (%)	50 (91)	4 (40)	0.001
No AF/AT within 3-month blanking period, *n* (%)	50 (91)	2 (20)	<0.001

Values are expressed as *n* (%) or medians [first and third quartiles]

*AF* atrial fibrillation, *AT* atrial tachycardia, *PV* pulmonary vein, *NT-proBNP* N-terminal prohormone of brain natriuretic peptide

Perioperative complications occurred in 4 (6.2%) patients: transient diaphragmatic nerve injury during RSPV ablation (recovered intraoperatively), stroke in the left middle cerebral artery territory (recovered to the point where the patient could walk with a cane 3 months later), stroke in the left cerebellum (symptoms completely resolved 6 days after ablation), and postoperative prolonged hypotension with decreased left ventricular ejection fraction (LVEF, 22%) that required 2 days in intensive care unit (LVEF improved to 53% after 6 months) in 1 patient each. No esophagus-related complications, such as gastric hypomotility and atrial-esophageal fistula were observed in any patient.

Of the 10 patients with recurrent arrhythmias after ablation, 5 (all of whom had complete roof block the first procedure) underwent a second catheter ablation. The results of the second catheter ablation are summarized in Online Resource 2. Of the two patients who had recurrence as AT, one was found to have recurrence of roof line conduction and was diagnosed with roof-dependent AT.

### Predictors of ablation success

**Fig. 3:**
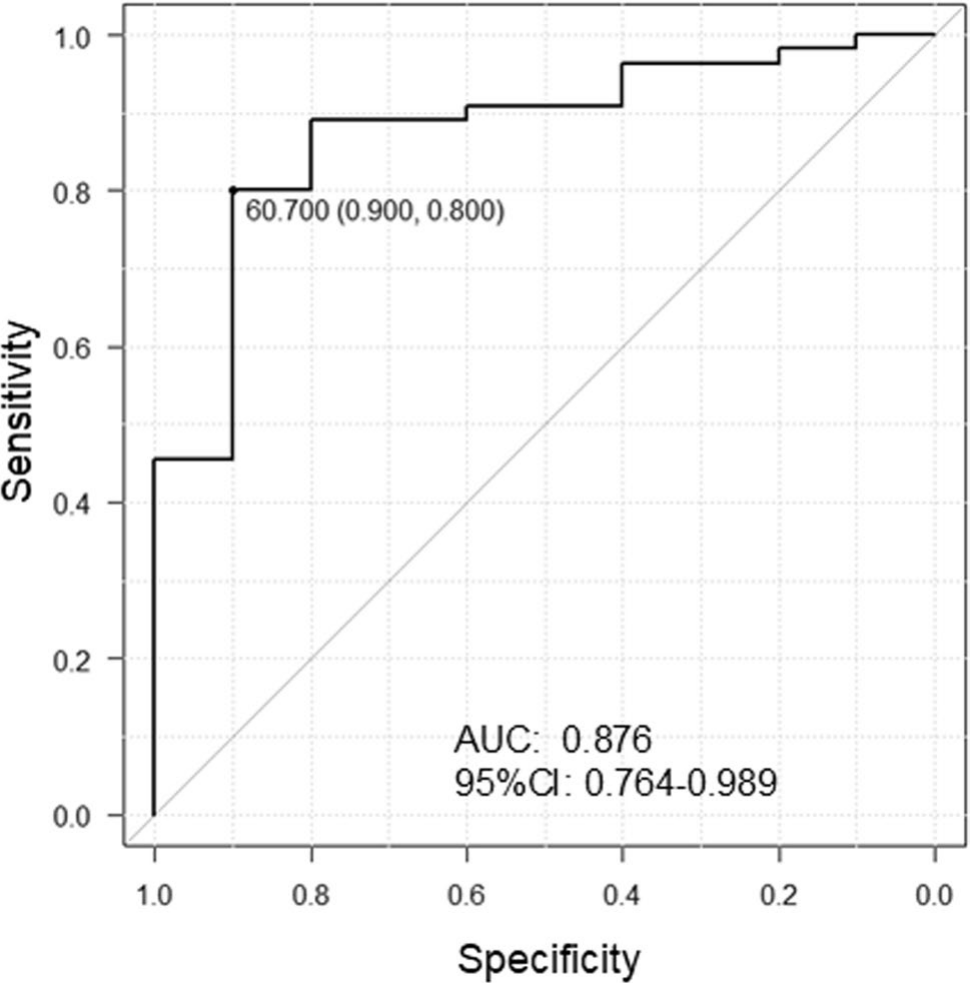
Receiver operating characteristic (ROC) curve for no recurrence of atrial arrhythmias during 1-year post-ablation and the reduction rate of NT-proBNP level at 1-month post-ablation. The optimal cutoff value of the reduction rate of NT pro-BNP level at 1-month after ablation is 60.7% with a sensitivity of 80% and specificity of 90%. AUC, area under the curve; CI, confidence interval; NT-proBNP, N-terminal prohormone of brain natriuretic peptide

**Fig. 4:**
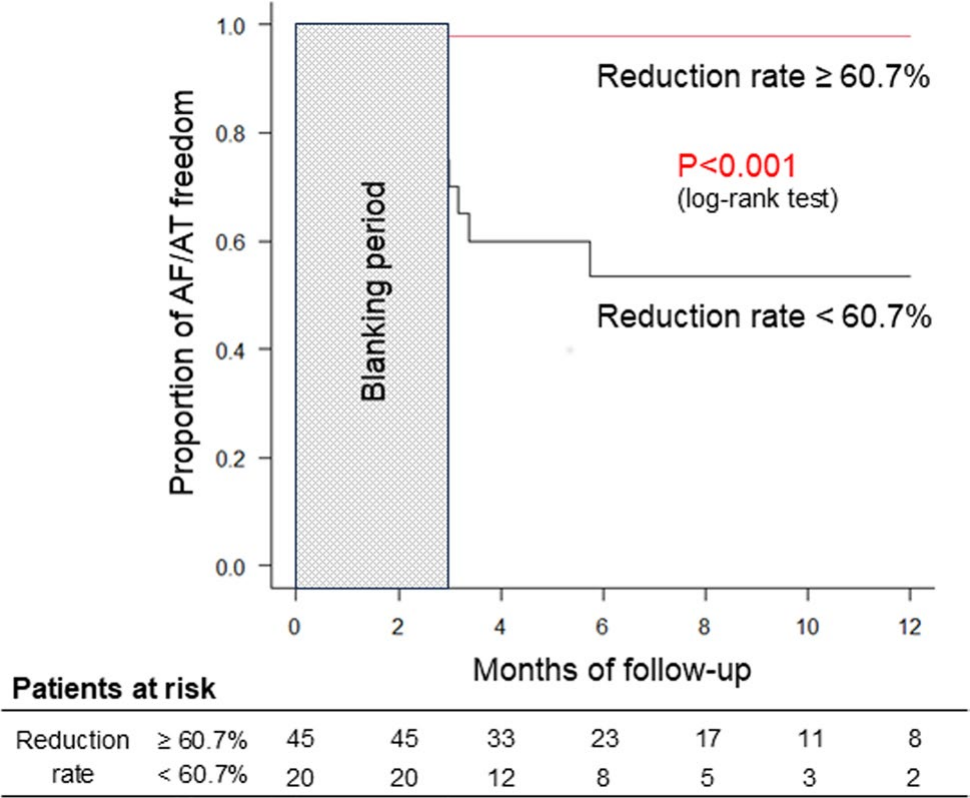
Kaplan–Meier curve of a single procedure atrial arrhythmia freedom after a cryoballoon ablation. *P* < 0.001 for the reduction rate of NT-proBNP level at 1-month post-ablation ≥60.7% vs. <60.7% is shown. AF, atrial fibrillation; AT, atrial tachycardia; CI, confidence interval; NT-proBNP, N-terminal prohormone of brain natriuretic peptide

In the multivariable model, the arrhythmia recurrence was significantly associated with longer AF duration (Model 1: odds ratio [OR], 1.120; 95% confidence interval [95% CI], 1.027–1.222; *P* = 0.011; Model 2: OR, 1.102; 95% CI, 1.010–1.202; *P* = 0.029), smaller ΔNT-proBNP_1 month_ (Model 1: OR, 0.937; 95% CI, 0.891–0.985; *P* = 0.010; Model 2: OR, 0.942; 95% CI, 0.894–0.991; *P* = 0.022), and no arrhythmia recurrence within the 3-month blanking period (Model 2: OR, 0.015; 95% CI, 0.000–0.986; *P* = 0.049) (Table 4). In the ROC curve, ΔNT-proBNP_1 month_ ≥ 60.7% predicted the ablation success at 1-year post-ablation with an AUC of 0.876 (*P* < 0.001; Fig. 3). ΔNT-proBNP_1 month_, sinus rhythm at 1-month post-ablation, and no arrhythmia (AF/AT) recurrence within the 3-month blanking period predicted ablation success at 1-year post-ablation with a high accuracy (Table 5). The cutoff value of ΔNT-proBNP_1 month_ for all patients was 60.7%, and Kaplan–Meier survival analysis showed that the arrhythmia-free rate at 1-year post-ablation was higher in patients with ΔNT-proBNP_1 month_ ≥ 60.7% than in those with ΔNT-proBNP_1 month_ < 60.7% (*P* < 0.001 by the log-rank test; Fig. 4).

**Table 4:** Multivariable model for risk of recurrence of atrial arrhythmia

	Model-1			Model-2		
	Odds ratio	95% confidence interval	*P* value	Odds ratio	95% confidence interval	*P* value
Age	1.020	0.888–1.171	0.782	1.001	0.854–1.172	0.994
Gender (Male reference)	0.123	0.003–5.098	0.270	0.268	0.006–11.981	0.497
Body mass index	1.295	1.295–1.968	0.226	1.433	0.866–2.374	0.162
AF duration	1.120	1.027–1.222	0.011	1.102	1.010–10202	0.029
Reduction rate of NT-proBNP at 1-month post-ablation	0.937	0.891–0.985	0.010	0.942	0.894–0.991	0.022
Sinus rhythm at 1-month post-ablation	0.033	0.001–1.248	0.066			
No recurrence (AF/AT) within 3-month blanking period				0.015	0.000–0.986	0.049

Model 1: Sinus rhythm at 1-month post-ablation is included as a variable. Model-2: No recurrence (AF/AT) within the 3-month blanking period is included as a variable

*AF* atrial fibrillation, *AT* atrial tachycardia, *NT-proBNP* N-terminal prohormone of brain natriuretic peptide

**Table 5:** Sensitivity, specificity, and predictive accuracy of the criteria for predicting no recurrence of atrial arrhythmia 1-year post-ablation

Criterion	Sensitivity, %	Specificity, %	Positive predictive value, %	Negative predictive value, %
Reduction rate of NT-proBNP at 1-month post-ablation ≥ 60.7% (maximum value of area under curve)	80.0	90.0	97.8	45.0
Reduction rate of NT-proBNP at 1-month post-ablation ≥ 75.6% (median value)	58.2	90.0	97.0	28.1
Reduction rate of NT-proBNP at 1-month post-ablation ≥ 35.6%	90.9	60.0	92.6	54.5
Sinus rhythm at 1-month post-ablation	90.9	60.0	92.6	54.5
No AF/AT within 3-month blanking period	90.9	80.0	96.2	89.2

The reduction rate of NT-proBNP level at 1-month post-ablation ≥35.6% predicts no arrhythmia recurrence at 1-year post-ablation, with the same accuracy as sinus rhythm at 1-month post-ablation

*AF* atrial fibrillation, *AT* atrial tachycardia, *NT-proBNP* N-terminal prohormone of brain natriuretic peptide

## Discussion

The main findings of the present study in 65 patients with PeAF undergoing LA roof ablation as well as PV isolation using a cryoballoon catheter are the following; (1) The success rate of complete block of the LA roof by cryoballoon ablation was high (92.3%), and there were no esophagus-related complications, (2) an LA volume < 87.4 mL may serve as a predictor for complete block of the LA roof, (3) the arrhythmia-free rate at 1-year post-ablation was 83.3%, and the ΔNT-proBNP_1 month_, but not the baseline NT-proBNP level, was a significant predictor, (4) arrhythmia recurrences after ablation were found more often within the 3-month blanking period, and no arrhythmia recurrence within the blanking period was also a significant predictor of no arrhythmia recurrence at 1 year, (5) ΔNT-proBNP_1 month_ ≥ 60.7%, sinus rhythm at 1-month follow up, and no AF/AT within the 3-month blanking period were highly predictive of no recurrence of arrhythmia at 1 year postoperatively.

### Efficacy of LA roof ablation in patients with PeAF

Catheter ablation for paroxysmal AF using a second-generation cryoballoon has demonstrated good mid- and long-term clinical outcomes. However, PV isolation alone may not be sufficient in patients with PeAF, as atrial fibrosis and remodeling progress with each AF episode [14, 15]. In fact, in the STOP Persistent AF trial, the 1-year recurrence-free atrial arrhythmia rate for PV isolation using a cryoballoon was low at 54% [8]. However, subsequent studies on LA roof ablation and PV isolation using a cryoballoon for patients with PeAF have demonstrated that the arrhythmia-free rate at 1-year post-ablation was 75.6–87.4% [16, 17], which was comparable with our result (arrhythmia-free rate at 1-year post-ablation, 83.3%). In this study, the success rate of complete block of the LA roof was 92.3%, which was also comparable to that reported in the previous studies (84.1–91.9%) [16, 17]. No esophagus-related complications were observed in this study. Based on these findings, we believe that LA roof ablation, in addition to PV isolation, is effective and feasible in patients with PeAF.

In this study, patients with an incomplete roof block had a greater number of cryoballoon applications and larger LA volume than those without an incomplete roof block (Table 2). An LA volume < 87.4 mL may be a predictor of complete roof block (Fig. 2). Furthermore, patients with an incomplete roof block tended to have longer AF duration, longer roofline length and height, and greater LA diameter and LA volume index than those without an incomplete roof block. These findings indicate that an incomplete roof block is more likely to occur in patients with advanced LA remodeling. Conversely, complete roof block creation using a cryoballoon might be relatively easy to achieve in patients with PeAF without advanced LA remodeling.

### Safety of LA roof ablation in patients with PeAF

In this study, 2 patients (3%) developed cerebral infarction after catheter ablation, both with a CHA_2_DS_2_-VASc score of 3 and a high NT-proBNP level. A high CHA_2_DS_2_-VASc score (≥2) is an independent risk factor for post-ablation cerebral infarction in patients with AF [18]. Furthermore, high BNP levels are associated with severe stroke, even in patients taking oral anticoagulants [19]. In our cohort, the CHA_2_DS_2_-VASc scores [17, 20] and NT-proBNP levels[21] were higher than those in similar studies on catheter ablation for PeAF, suggesting that this cohort may have had a slightly elevated risk of embolic events. For 1 patient with sequelae (stroke in the left middle cerebral artery territory), it is possible that oral administration of low-dose edoxaban was not sufficient for anticoagulation [22].

In this study, we could not determine whether these 2 stroke cases were due to LA roof ablation. Patients with PeAF tend to be older and have higher CHA_2_DS_2_-VASc scores than those with paroxysmal AF [23, 24]. Therefore, great caution should be exercised regardless of whether LA roof ablation is performed, especially in patients with PeAF with higher CHA_2_DS_2_-VASc scores.

Although late recurrence of the roof line conduction could not be systematically assessed in this study, one patient with recurrent AT was found to have roof line reconduction during the second procedure, and was diagnosed with roof-dependent AT. Therefore, although rare (1.5%), the possibility of iatrogenic AT due to roof line ablation cannot be completely excluded.

### Predictors of atrial arrhythmia recurrence

Recurrence of atrial arrhythmias after catheter ablation for AF has been associated with various predictive factors, including body mass index, LA diameter, LVEF, AF duration, CHA_2_DS_2_-VASc score, and imaging findings such as epicardial fat, left atrial and left atrial appendage volume on CT, and fibrosis on magnetic resonance imaging [11]. In addition, recent studies have reported that pretreatment BNP levels may influence prognosis [10]. Moreover, early recurrence within the 3-month blanking period has also been suggested to impact long-term outcomes [25]. In cryoballoon ablation of AF, early recurrence after ablation was also associated with a high risk for late recurrence [26]. Furthermore, limiting the blanking period to 1 month after cryoballoon ablation has been proposed [27].

This is the first study to examine the predictors of arrhythmia recurrence in patients with PeAF who underwent LA roof ablation and PV isolation. Shorter duration of AF, no recurrence of AF/AT within the 3-month blanking period, and higher ΔNT-proBNP_1 month_ were identified as significant predictors of no arrhythmia recurrence during 1-year post-ablation. In this study, arrhythmia recurrence was often observed early after ablation, as was the case with PV isolation ablation in patients with PeAF. However, AF/AT recurrence at 1-month post-ablation was not a significant predictor of the absence of arrhythmia recurrence during the 1-year post-ablation period. However, ΔNT-proBNP_1 month_, but not pre-ablation BNP levels, was a significant predictor of arrhythmia recurrence during 1-year post-ablation. Previous studies have reported that pre-ablation BNP and NT-proBNP levels are associated with long-term recurrence after ablation, including cryoballoon ablation of AF and that lower values are associated with no arrhythmia recurrence, especially in paroxysmal AF [10, 28]. However, in patients with PeAF, pre-ablation BNP levels are higher than those with paroxysmal AF [10, 29] and do not correlate with recurrence [10]. Similarly, in our study, pre-ablation NT-proBNP levels were not associated with arrhythmia recurrence at 1-year post-ablation. In patients with PeAF, BNP/NT-proBNP levels are elevated before ablation because of AF-induced hemodynamic loading and remodeling [30], and pre-ablation BNP/NT-proBNP levels may not accurately predict recurrent arrhythmias. Conversely, in this study, NT-proBNP level at 1-month post-ablation was much lower in patients without arrhythmia recurrence during 1-year post-ablation than in those with a recurrence, and the ΔNT-proBNP_1 month_ was a significant predictor of arrhythmia recurrence at 1-year post-ablation. Restoration of sinus rhythm after AF ablation reduced the AF-induced hemodynamic load, and reverse structural remodeling might be the reason for decreased NT-proBNP levels at 1-month post-ablation in patients without arrhythmia recurrence.

In this study, ΔNT-proBNP_1 month_ and no AF/AT within the 3-month blanking period were significant predictors of arrhythmia recurrence during 1-year post-ablation, and both predicted arrhythmia recurrences with high accuracy (Tables 4 and 5). Arrhythmia detection during the 3-month blanking period with periodic 12-lead ECG recordings and implantable cardiac monitors may be difficult. In contrast, ΔNT-proBNP_1 month_ can be measured at 1-month post-ablation and can predict arrhythmia recurrences with high accuracy. We believe that ΔNT-proBNP_1 month_ may serve as a simple and potentially useful predictor in patients with PeAF undergoing LA roof ablation and PV isolation.

In this study, 14 patients (22%) were on SGLT2 inhibitors prior to ablation, and this number increased to 15 patients (23%) at the 1-month follow-up. Loop diuretics were used by 10 patients (15%) before ablation, decreasing to 9 patients at follow-up, while the use of other diuretics remained unchanged. Given these minimal changes, the influence of concomitant medications on NT-proBNP levels is considered to be limited.

In this study, 18 patients (27.7%) underwent additional RF ablation beyond PV isolation and LA roof ablation, based on the clinically documented or inducible atrial tachyarrhythmias. As shown in Table 2, the presence of this additional ablation was not significantly associated with arrhythmia recurrence. Although the present study did not evaluate healthcare costs, the question of economic efficiency remains important when comparing ablation strategies. Given that nearly 30% of patients required additional ablation, future prospective studies are warranted to compare the cost-effectiveness of a stepwise approach—beginning with cryoballoon PV isolation and LA roof ablation followed by selective RF ablation as needed—versus a primary RF ablation strategy. Such investigations would help determine whether procedural tailoring based on arrhythmia inducibility provides both clinical and economic benefit.

### Limitations

This study has several limitations. First, it was a single-center single-arm observational study with a relatively small cohort. Second, the use of antiarrhythmic drugs during follow-up was discretion of the treating physician and was not standardized. Third, the incidence of ischemic stroke was higher than that previously reported [31], potentially due to a higher CHA₂DS₂-VASc score and NT-proBNP level in this patient cohort compared with other AF ablation groups. In addition, one patient had a history of epistaxis, which may have necessitated the use of a low dose of anticoagulants, possibly contribution to thromboembolic complications.

Furthermore, the complete LA roof line was confirmed using voltage and activation mapping during atrial pacing at the distal coronary sinus; however, differential pacing within the LA was not performed to validate conduction block.

Finally, this study did not assess the cost-effectiveness of different ablation strategies, which may be important for future clinical decision making.

### Conclusions

The present study demonstrated that the success rate of complete block of the LA roof by cryoballoon ablation and the arrhythmia-free rate at 1-year post-ablation were high, without esophagus-related complications. We suggest that ΔNT-proBNP at 1-month post-ablation may serve as a simple and potentially useful predictor in patients with PeAF undergoing LA roof ablation and PV isolation; however, further validation in larger, prospective studies is warranted.

## Supplementary Information

Below is the link to the electronic supplementary material.

Supplementary file1 (PDF 259 KB)
