# Supplementary material for: Impact of NT-proBNP reduction on recurrence after cryoballoon pulmonary vein isolation and left atrial roof ablation in persistent atrial fibrillation
Source: Heart Vessels. 2025 Jun 6;40(11):1017–26. doi: 10.1007/s00380-025-02559-x (PMC12532725; doi:10.1007/s00380-025-02559-x)
Supplement: Supplementary file 1 — Supplementary file1 (PDF 259 KB) [file 380_2025_2559_MOESM1_ESM.pdf]

Heart and Vessels:

Impact of NT-proBNP Reduction on Recurrence After Cryoballoon Pulmonary Vein Isolation and Left Atrial Roof Ablation in Persistent Atrial Fibrillation

Ryohei Nomura, MD; Kanae Hasegawa, MD, PhD; Toshihiko Tsuji, MD; Moe Mukai, MD, PhD; Machiko Miyoshi, MD, PhD; Naoto Tama, MD, PhD; Hiroyuki Ikeda, MD, PhD; Kentaro Ishida, MD, PhD; Hiroyasu Uzui, MD, PhD; Hiroshi Tada, MD, PhD.

Department of Cardiovascular Medicine, Faculty of Medical Sciences, University of Fukui, Fukui, Japan

Address for correspondence: Kanae Hasegawa, MD, PhD

Department of Cardiovascular Medicine, Faculty of Medical Sciences, University of Fukui

23-3, Matsuokashimoaizuki, Eiheiji-cho, Yoshida-gun, Fukui 910-1193, Japan

E-mail: kanaeh@u-fukui.ac.jp Phone: +81-776-61-8800 FAX: +81-776-61-8801

Online Resource 1. Technique of left atrial roof ablation using a cryoballoon.

Representative anteroposterior fluoroscopic images and the cryoballoon position from right to left are shown.

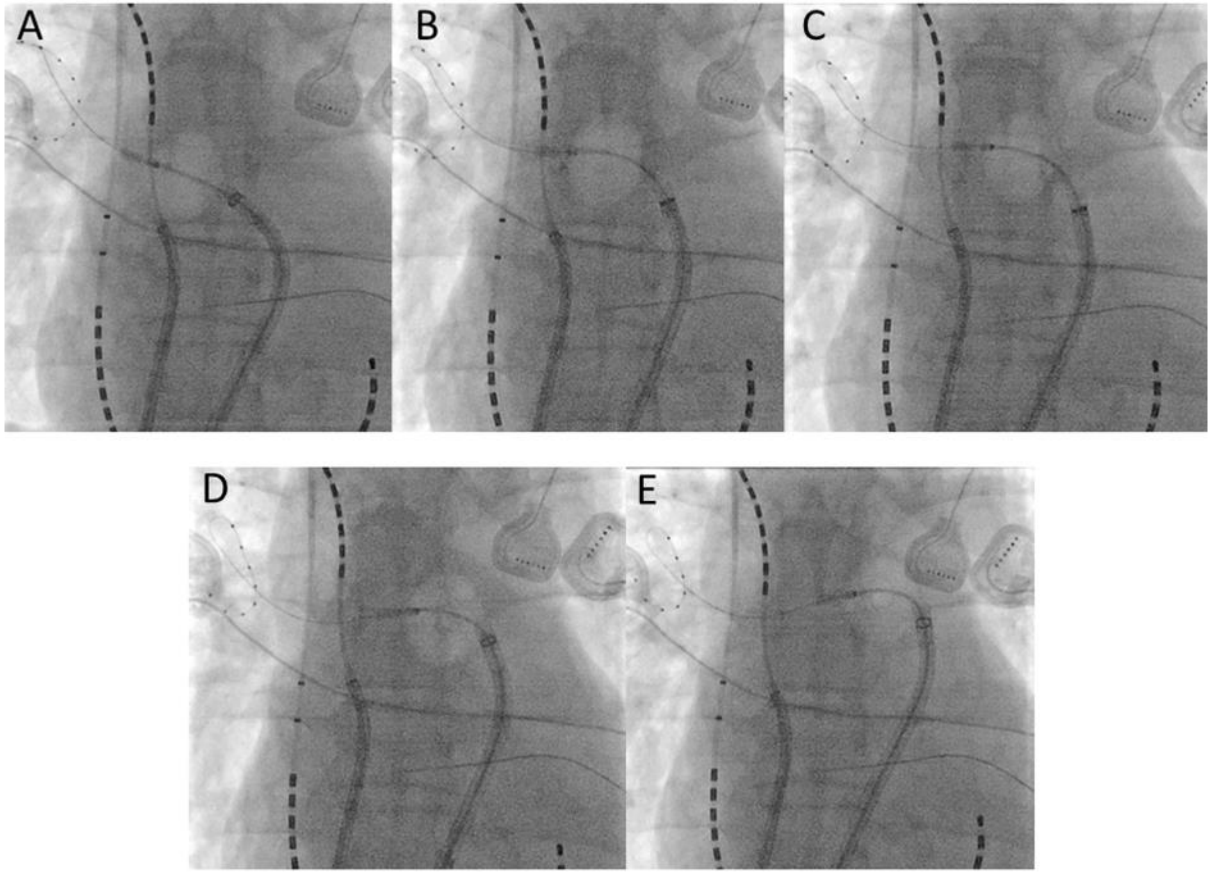

Online Resource 2. Patients with recurrences undergoing a second catheter ablation

Pt	Age, years	Gender	AF duration, months	1st ablation		Recurrence		2nd ablation		
				Roof line with block	Additional ablation	Rhythm	Date (first recurrence day)	Recurrence of PV conduction	Recurrence of roof line conduction	Treatment
1	81	Male	19	complete	CTI linear ablation	AT	within blanking period (42 days)	Left and right superior PVs	(+)	LA roof ablation with complete conduction block for roof-dependent AT
2	82	Male	37	complete		AF	within blanking period (40 days)	Right inferior PV	(+)	LA box isolation: PV isolation, roof ablation with complete conduction block, bottom line ablation with complete conduction block; CTI linear ablation
3	60	Male	36	complete		AT	within blanking period (46 days)	(-)	(-)	Mitral isthmus linear ablation with complete conduction block for mitral isthmus-dependent AT; CTI linear ablation
4	55	Male	170	complete		AF	within blanking period (1 day)	(-)	(-)	LA box isolation: bottom line ablation with complete conduction block; Mitral isthmus linear ablation with complete conduction block; CTI linear ablation

5	68	Male	23	complete	CTI linear ablation	AF	within blanking period (48 days)	(-)	(-)	LA box isolation: bottom line ablation with complete conduction block; SVC isolation; CTI linear ablation for the conduction recurrence

AF, atrial fibrillation; AT, atrial tachycardia; CTI, cavotricuspid isthmus; LA, left atrium; Pt, patient; PV, pulmonary vein; SVC, superior vena cava.
